# P2X7 Receptor Induces Pyroptotic Inflammation and Cartilage Degradation in Osteoarthritis via NF-*κ*B/NLRP3 Crosstalk

**DOI:** 10.1155/2021/8868361

**Published:** 2021-01-16

**Authors:** Zihao Li, Ziyu Huang, He Zhang, Jinghan Lu, Yicheng Tian, Yingliang Wei, Yue Yang, Lunhao Bai

**Affiliations:** ^1^Department of Orthopedic Surgery, Shengjing Hospital of China Medical University, Shenyang 110000, China; ^2^Foreign Languages College, Shanghai Normal University, Shanghai 200234, China

## Abstract

Osteoarthritis (OA) is an urgent public health problem; however, the underlying causal mechanisms remain unclear, especially in terms of inflammatory mediators in cartilage degradation and chondrocyte imbalance. P2X7 receptor (P2X7R) is a critical inflammation switch, but few studies have examined its function and mechanisms in OA-like pyroptotic inflammation of chondrocytes. In this study, Sprague–Dawley rats were injected in the knee with monosodium iodoacetate (MIA) to induce OA, followed by multiple intra-articular injections with P2X7R antagonist A740003, P2X7R agonist BzATP, NF-*κ*B inhibitor Bay 11-7082, and NLRP3 inhibitor CY-09. Primary rat chondrocytes were harvested and treated similarly. We assessed cell viability, damage, and death via cell viability assay, lactate dehydrogenase (LDH) release, and flow cytometry. Concentrations of adenosine triphosphate (ATP) and interleukin- (IL-) 1*β* in cell culture supernatant and joint cavity lavage fluid were analyzed by enzyme-linked immunosorbent assay. Changes in expression levels of P2X7 and inflammation-related indicators were analyzed by immunofluorescence, quantitative reverse-transcription polymerase chain reaction, and western blotting. Cell morphology changes and pyroptosis were observed using transmission electron microscopy. Histology, immunohistochemistry, and microcomputed tomography were used to analyze damage to bone and cartilage tissues and assess the severity of OA. Similar to MIA, BzATP reduced cell viability and collagen II expression in a dose-dependent manner. Conversely, A740003 ameliorated MIA-induced cartilage degradation and OA-like pyroptotic inflammation by rescuing P2X7, MMP13, NF-*κ*B p65, NLRP3, caspase-1 (TUNEL-positive and active), and IL-1*β* upregulation. Additionally, A740003 reduced the caspase-1/propidium iodide double-positive rate, LDH concentration, and reactive oxygen species production. These effects also occurred via coincubation with Bay 11-7082 and CY-09. In conclusion, activated P2X7 promoted extracellular matrix degradation and pyroptotic inflammation in OA chondrocytes through NF-*κ*B/NLRP3 crosstalk, thus, aggravating the symptoms of OA. The study findings suggest P2X7 as a potential target for inflammation treatment, providing new avenues for OA research and therapy.

## 1. Introduction

Osteoarthritis (OA) is a degenerative joint disease, whose clinical features include articular cartilage loss, synovitis, subchondral bone sclerosis, and osteophyte formation [1]. Aggravation of OA causes physical pain and inconvenience to patients, which severely diminishes their quality of life. Therefore, preventing and alleviating the occurrence and development of OA is one of the most urgent health problems in today's society [2]. The etiology of OA is complex and multifactorial. From a macroscopic point of view, factors such as aging, strain, and trauma affect the synovium and cartilage [3]. From a microscopic perspective, a breakdown in the metabolic balance of joint cells leads to exuberant expression of proinflammatory molecules and changes in the cartilage matrix microenvironment [4]. Thus, understanding the cellular and molecular mechanisms involved in the release of inflammatory mediators and cartilage degradation has important implications for OA treatment.

As the most crucial structural part of the knee joint, cartilage directly affects OA development and progression. Type II collagen combines with other collagen and noncollagen proteins to form a stable meshwork that provides tensile strength for cartilage [5]. As the only cell type in cartilage, chondrocytes synthesize and are wrapped in extracellular matrix. Chemical and mechanical changes in the cellular environment strictly control the structural and biochemical components of cartilage [6]. Therefore, OA is linked to chondrocyte condition and catabolism of the extracellular matrix. Transcription factor nuclear factor- (NF-) *κ*B plays a key role in inflammation. Activation of the NF-*κ*B pathway inhibits chondrocyte anabolism and induces the expression of matrix-degrading proteases, such as matrix metalloproteases (MMPs) and a disintegrin and metalloproteinase with thrombospondin motifs (ADAMTS) [7]. In addition, NF-*κ*B activation mediates OA-chondrocyte production of catabolic cytokines and chemokines, including tumor necrosis factor- (TNF-) *α*, interleukin- (IL-) 1*β*, and receptor activator NF-*κ*B ligand (RANKL). These molecules reduce collagen synthesis and augment NF-*κ*B signaling through a positive feedback loop [8].

The purinergic receptor P2X7 receptor (P2X7R), encoded by the *P2RX7* gene, is highly relevant to inflammation. Functional ion channels of P2X7R subunits consist of stable trimers in the plasma membrane [9]. Upon adenosine triphosphate (ATP) binding, P2X7 gates open to support Na^+^ and Ca^2+^ influx, coupled with K^+^ efflux, resulting in rapid depolarization. P2X7 can also be activated by BzATP, an ATP analog with greater potency than ATP. P2X7-dependent signaling pathways involve cytokine release. For example, K^+^ efflux is an essential upstream event for activating the NLRP3 inflammasome, proteases (e.g., caspases, cathepsins, and MMPs), and transcription factors (e.g., NF-*κ*B p65, HIF-1*α*, and PI3K-AKT-GSK-3*β*). Overall, P2X7 regulates a series of signaling pathways involved in inflammation, including ion flow, protease activation, membrane response, and secretion [10]. Understanding P2X7 function can provide insights into OA development and progression.

As a key inflammation switch, P2X7 mediates the main downstream response to activate inflammasomes, which are central to pyroptosis and comprise receptor protein NLRP3, adaptor protein ASC, and pro-caspase-1. NLRP3 activates and cleaves caspase-1 to cause secretion of bioactive IL-1*β*, leading to an inflammatory response [11, 12]. NLRP3 activation occurs in two ways. First, the NF-*κ*B signaling pathway increases pro-IL-1*β* and NLRP3 synthesis. Second, oligomerization is initiated to assemble the NLRP3 inflammasome [13]. Opening P2X7 channels or membrane pores alters the local ionic microenvironment in cells, driving the recruitment and assembly of inflammasome components [14]. However, whether P2X7 can induce pyroptotic inflammation in chondrocytes to aggravate OA is unknown. We hypothesized that activated P2X7R in chondrocytes promotes extracellular matrix degradation and IL-1*β* release via NF-*κ*B/NLRP3 crosstalk, subsequently aggravating OA development and progression.

The goal of the study was to examine P2X7 function and mechanisms in OA-like pyroptotic inflammation of chondrocytes. Intra-articular monosodium iodoacetate (MIA) injection is commonly employed to create OA rat models; however, its ability to induce an OA-like phenotype in chondrocytes is unclear [15]. To the best of our knowledge, this study is the first to investigate whether MIA stimulation changes the chondrocyte phenotype, specifically in terms of extracellular ATP and P2X7 expression. We also examined the effects of a P2X7R antagonist, agonist, and pathway inhibitors on P2X7 expression and activation to explore the underlying causal mechanisms of OA. Collectively, our findings may offer a fresh perspective on therapeutic strategies for OA.

## 2. Materials and Methods

### 2.1. mRNA-Sequencing Data

Dataset GSE114007 [13] was retrieved from the Gene Expression Omnibus (GEO) database to determine whether *P2X7* was differentially expressed in humans with OA. RNA-sequencing identified genes, pathways, and regulatory networks dysregulated in patients with OA. Further analysis used sample data from 10 healthy individuals and 10 patients with OA from the GEO database (https://www.ncbi.nlm.nih.gov/geo) [16, 17].

### 2.2. Antibodies and Reagents

The following antibodies were employed for western blotting: anti-P2X7 (Abcam, Cambridge, UK), anti-collagen II (Abcam), anti-MMP13 (Abcam), anti-IL-1*β* (Abcam), anti-NF-*κ*B p65 (Cell Signaling Technology (CST), Danvers, MA, USA), anti-p-NF-*κ*B p65 (CST), anti-NLRP3 (Proteintech Group, Chicago, IL, USA), anti-caspase-1 (Proteintech Group), anti-GAPDH (Proteintech Group), and HRP-labeled IgG (Beyotime Biotech, Shanghai, China). Primary chondrocyte cultures were treated with MIA (Sigma-Aldrich, St. Louis, MO, USA), P2X7R antagonist A740003 (Sigma-Aldrich), P2X7R agonist BzATP (Sigma-Aldrich), NF-*κ*B inhibitor Bay 11-7082 (Sigma-Aldrich), and NLRP3 inhibitor CY-09 (Sigma-Aldrich).

### 2.3. Animals, OA Induction, and Knee-Joint Injection

Sixty male Sprague–Dawley (SD) rats (6 weeks old, 230–250 g) were purchased from HFK Bioscience (Beijing, China). With a controlled temperature of 22 ± 2°C and 70% humidity, rats were housed with 12 h of light/dark cycles and fed with adequate food and drinking water. All experiments were performed in compliance with institutional guidelines and approved by the Committee of the China Medical University (no. 2017PS237K). During experiments, we adhered to the 3R rules to ensure that rats were sacrificed comfortably. Rats were anesthetized with sodium pentobarbital and randomly divided into five groups: (1) saline, (2) MIA, (3) MIA+A740003, (4) MIA+BzATP, and (5) MIA+BzATP+Bay 11-7082+CY-09. To induce OA, rats were injected once in the knee with MIA (0.6 mg/knee) dissolved in 50 *μ*L saline, using a 30-gauge needle during the first week. A740003, Bay 11-7082, and CY-09 were dissolved in DMSO. MIA-injected rats were then given multiple 50 *μ*L intra-articular injections of saline, A740003 (0.6 mg/knee), BzATP (0.3 mg/knee), Bay 11-7082 (0.3 mg/knee), and CY-09 (0.3 mg/knee) twice weekly for 4 weeks, as previously described [18].

### 2.4. Extraction of Rat Tissue

Rats were euthanized via a sodium pentobarbital overdose at 12 weeks to extract knee-joint tissues. For staining, morphological analysis, and immunohistochemistry, knee-joints were fixed with 4% paraformaldehyde for 48 h and decalcified in 10% EDTA for 4 weeks before being embedded in paraffin. Intra-articular lavage fluid (IALF) was collected from the knee synovial cavity and immediately used for subsequent experiments.

### 2.5. Primary Chondrocyte Cultures and Experimental Design

Cartilage samples from the tibial plateau and femoral heads of the rats were harvested and digested for 30 min with protease K (4 mg/mL, Roche, Basel, Switzerland), followed by a 2 h digestion with collagenase D (1.6 mg/mL, Roche) at 37°C. After digestion, the cell suspensions were centrifuged for 5 min at 1200 rpm. Primary chondrocytes were plated in culture flasks and maintained in Dulbecco's Modified Essential Medium: nutrient mixture F-12 (DMEM/F-12) (Hyclone, Thermo Fisher Scientific, Waltham, MA, USA) containing 10% fetal bovine serum (FBS; Gibco, Thermo Fisher Scientific) and 1% penicillin/streptomycin (Thermo Fisher Scientific) at 37°C and under 5% CO_2_ atmosphere. Cells (primary passage = 3) were used for experiments. To determine dose- and time-dependence, chondrocytes were plated in 96-well plates with various doses of MIA (0, 0.75, 1.5, and 3 *μ*M), A740003 (0, 10, 20, and 50 *μ*M), and BzATP (0, 10, 50, and 100 *μ*M) for different durations (12 and 24 h). Cell viability was measured using the Cell Counting Kit-8 (CCK-8) assay.

For other experiments, chondrocytes were plated in T25 flasks or six-well plates and divided into eight groups: control (DMEM/F-12 only), MIA (DMEM/F-12 with 1.5 *μ*M MIA for 12 h), A740003 (DMEM/F-12 with 20 *μ*M A740003 for 12 h), MIA+A740003 (DMEM/F-12 with 20 *μ*M A740003 for 1 h, followed by 1.5 *μ*M MIA for 12 h), BzATP (DMEM/F-12 with 50 *μ*M BzATP for 12 h), MIA+BzATP (DMEM/F-12 with 50 *μ*M BzATP for 1 h, followed by 1.5 *μ*M MIA for 12 h), MIA+BzATP+Bay 11-7082 (DMEM/F-12 with 50 *μ*M BzATP for 1 h, followed by 1.5 *μ*M MIA and 10 *μ*M Bay 11-7082 for 12 h), and MIA+BzATP+CY-09 (DMEM/F-12 with 50 *μ*M BzATP for 1 h, followed by 1.5 *μ*M MIA and 10 *μ*M CY-09 for 12 h).

### 2.6. Cell Viability Assays

After treatment, 5 × 10^3^ chondrocytes/well were seeded in 96-well plates and incubated at 37°C and under 5% CO_2_ atmosphere for 12 or 24 h. At both time points, 10 *μ*L CCK-8 (Beyotime) and 90 *μ*L DMEM/F-12 were added to each well. After 2 h, a Synergy H1 microplate reader (BioTek Instruments Inc., Winooski, VT, USA) was used to determine sample absorbance at 450 nm.

### 2.7. Western Blotting

After the treated chondrocytes reached 80% confluence, they were lysed with radioimmunoprecipitation assay (RIPA) buffer containing phenylmethyl sulfonylfluoride (PMSF) and protease/phosphatase inhibitors (Beyotime Biotech). Protein concentrations were measured using a Bicinchoninic Acid (BCA) Protein Assay Kit (Beyotime Biotech). Protein mixtures (20 *μ*g/lane) were separated using sodium dodecyl sulfate–polyacrylamide gel electrophoresis (SDS-PAGE) and transferred onto polyvinylidene difluoride (PVDF) membranes (GE, Thermo Fisher Scientific). Membranes were blocked with 5% nonfat dry milk for 2 h at room temperature and incubated overnight with primary antibodies (1 : 1000) at 4°C. To control protein loading, the membranes were probed with anti-GAPDH antibody (1 : 2000) and incubated with HRP-labeled IgG antibodies (1 : 5000) for 2 h at room temperature. Results were scanned using chemiluminescence detection with the Amersham Imager 600 (GE Healthcare, Logan, UT, USA). Densitometry analysis of band intensities was performed using the ImageJ software (National Institutes of Health (NIH), Bethesda, MD, USA). The relative protein level was normalized to that of GAPDH.

### 2.8. RNA Isolation and qRT-PCR

Total RNA was extracted using RNAiso Plus (Takara Bio, Kusatsu, Japan). Purity was assessed through measurement of the A260/A280 ratio. Complementary DNA was synthesized using Prime Script™ RT Master Mix (TaKaRa Bio). Quantitative reverse-transcription PCR (qRT-PCR) was performed using the 7500 Real-Time PCR System (Applied Biosystems, Foster City, CA, USA) with TB Green^@^ Premix Ex Tag™ II (TaKaRa Bio). The thermocycling conditions were as follows: 95°C for 30 s, 40 cycles at 95°C for 5 s, and 60°C for 35 s. The housekeeping gene was GADPH. Relative gene expression was calculated using 2^−*ΔΔ*Ct^. The primer sequences are listed in Supplementary Figure S2.

### 2.9. Flow Cytometry

Inflammatory chondrocyte death was assessed using the caspase-1 fluorescent inhibitor probe FLICA 660-YVAD-FMK. After removing the media and washing three times, cells with membrane pores were marked with propidium iodide (PI) and measured using a FACSCalibur flow cytometer (BD Biosciences, San Jose, CA, USA). The double-positive rate was normalized to the control, and data were analyzed using the FlowJo software (BD Biosciences).

### 2.10. Immunofluorescence and Imaging Analysis

Chondrocytes were plated on cover-slipped cell-culture slides, fixed with 4% paraformaldehyde for 10 min, and permeabilized with 0.25% Triton X-100 at room temperature for 10 min. After washing with phosphate-buffered saline (PBS), cells were blocked with 5% goat serum for 30 min at room temperature and then incubated with primary antibodies (1 : 100) overnight at 4°C. After washing with PBS, the cells were stained with secondary antibodies (1 : 200) for 1 h and mounted in 4′,6-diamidino-2-phenylindole (DAPI) for 5 min at room temperature. Immunofluorescence images were obtained using a LSM880 confocal microscope (Zeiss, Oberkochen, Germany) and processed using the ImageJ software (NIH).

### 2.11. Cellular Reactive Oxygen Species (ROS) Production

The ROS Assay Kit (Beyotime Biotech) was used to determine cellular ROS production. Briefly, 2′,7-dichlorodihydrofluorescein diacetate (DCFH-DA was diluted with serum-free culture medium (1 : 1000) to a final concentration of 10 *μ*M/L. The cell culture fluid was removed, and diluted DCFH-DA was added in a sufficient volume to cover the cells, followed by incubation for 20 min at 37°C. The cells were washed three times with serum-free cell culture medium to fully remove the DCFH-DA that had not entered the cells. Direct observation was performed using a LSM880 confocal microscope (Zeiss, Oberkochen, Germany) (488 nm, excitation; 525 nm, emission).

### 2.12. Enzyme-Linked Immunosorbent Assay (ELISA)

After removal of cells and debris via centrifugation, the supernatant was collected and used immediately for subsequent experiments or stored at −80°C. The Rat IL-1 beta/IL-1F2 Quantikine ELISA Kit (R&D Systems, Minneapolis, MN, USA) was used to measure the quantity of IL-1*β* protein secreted into the cell culture supernatant and knee IALF, according to the manufacturer's instructions. Sample absorbance was measured at 450 nm within 30 min.

### 2.13. Lactate Dehydrogenase (LDH) Release and ATP Assay

LDH release was quantified in the cell culture supernatant using the LDH Cytotoxicity Assay Kit (Beyotime Biotech), according to the manufacturer's instructions. Briefly, appropriate treatments were added according to the experimental needs when the cell density reached 80–90%. One hour before the scheduled detection time point, LDH release reagent was added to the sample, mixed by pipetting several times, and incubated. After reaching the predetermined time, the cell culture plate was centrifuged at 400 g for 5 min. An aliquot of supernatant (120 *μ*L) was used to measure absorbance at 590 nm and 680 nm using a Synergy H1 microplate reader (BioTek Instruments). The fold increase in LDH concentration was normalized to the control.

ATP concentrations in the cell culture supernatant were determined using the Luminescent ATP Detection Assay Kit (Abcam) and Colorimetric/Fluorometric ATP Assay Kit (Abcam) following manufacturer protocols. Luminescence and Colorimetric/fluorometric absorbance were measured using a Synergy H1 microplate reader (BioTek Instruments Inc., Winooski, VT, USA).

### 2.14. Microcomputed Tomography (Micro-CT)

Rat knee joints were scanned using a SkyScan 1276 Micro-CT (Bruker, Kontich, Belgium) and NRecon version 1.6 software (Bruker). Data were then analyzed using the CTAn version 1.9 software (Bruker), and a three-dimensional model was generated in CTVol version 2.0 (Bruker). Quantitative morphometry indexes based on three-dimensional morphometry were determined using microtomographic data. Bone volume (BV), BV/total tissue volume (BV/TV), trabecular number (Tb.N), trabecular thickness (Tb.Th), and trabecular separation (Tb.Sp) were determined for the region of interest between the proximal tibia growth plate and tibial plateau.

### 2.15. Transmission Electron Microscopy (TEM)

The medium was discarded from cultured cells and cells were fixed in 2.5% glutaraldehyde for 5 min at room temperature, protected from light. Cells were then gently scraped in one direction, and the cell liquid was collected and centrifuged at 1000 g for 2 min. After discarding the fixative, 1% OsO_4_ was added, and the cell cluster was gently lifted and suspended. The cells were fixed for 30 min at room temperature, protected from light, then transferred and stored at 4°C. After dehydration, samples were embedded in resin. Ultrathin tissue sections (60 nm) were stained with uranyl acetate and lead citrate, and cell morphology and subcellular structures were observed using Hitachi 800 TEM equipment (Tokyo, Japan).

### 2.16. Histological Analysis, TUNEL Staining, and Immunohistochemistry (IHC)

Cartilage specimens were fixed in 4% paraformaldehyde for 48–72 h, decalcified in 10% ethylenediaminetetraacetic acid (EDTA) for 4–6 weeks, and embedded in paraffin. Each sample was cut into 4 *μ*m sections and then stained with hematoxylin and eosin (H&E) and Toluidine Blue O cartilage staining solution (Solarbio, Beijing, China) to observe histological and proteoglycan changes. After the sections were deparaffinized, antigen retrieval was performed using IHC Enzyme *Antigen Retrieval* Reagent (Boster Bio, Pleasanton, CA, USA). The universal two-step detection kit (ZSGB Biotech, Beijing, China) was then used to remove peroxidase according to the manufacturer's instructions. Sections were blocked with goat serum and then incubated with primary antibody overnight at 4°C. The following day, sections were incubated with secondary antibody, and finally, DAB staining and hematoxylin staining were performed. Sections were then dehydrated, mounted with neutral gum, and observed and recorded using an optical microscope. Two blinded observers used the Osteoarthritis Research Society International (OARSI) grading system to score cartilage destruction in the knee joints.

Sections were also stained using the TUNEL Apoptosis Detection Kit (Orange Fluorescence) (Abbkine Scientific Co., Ltd., Wuhan, China). After antigen retrieval, sections were permeabilized with 0.1% Triton X-100 at room temperature for 10–30 min and then incubated with the reaction mixture overnight at 4°C. The following day, sections were counterstained with DAPI and then observed and recorded using an Eclipse E800 fluorescence microscope (Nikon, Tokyo, Japan). The cell count, staining intensity, and immune-positive rate were calculated.

### 2.17. Statistical Analysis

All experiments were performed at least three times. Data are presented as means ± standard deviations (SD). Between-group differences were determined using Student's *t*-test or one-way ANOVA with Tukey's post hoc test with GraphPad Prism version 7.0 (GraphPad Software, La Jolla, CA USA). A *p* value < 0.05 was considered statistically significant.

## 3. Results

### 3.1. P2X7 Is Activated Concurrently with Pyroptotic Inflammatory Response in MIA-Exposed Chondrocytes

Screening of GEO data (Figure S1) indicated that *P2X7* was a differentially expressed gene significantly upregulated in OA compared with that in healthy human knee cartilage (*n* = 10).

**Figure 1:**
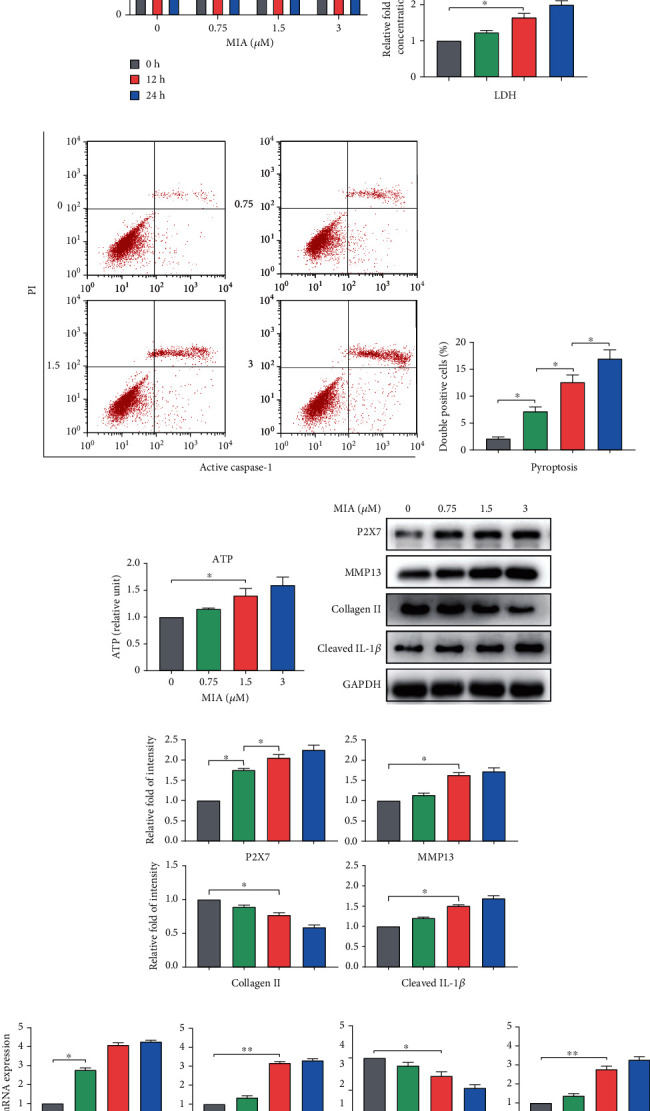
MIA induces inflammation and increases P2X7 expression. (a) Cell viability after chondrocytes were treated with different concentrations (0, 0.75, 1.5, and 3 *μ*M) of MIA for 12 or 24 h. (b) Histogram displaying LDH concentrations normalized to the control group. Grey, 0 *μ*M MIA; green, 0.75 *μ*M MIA; red, 1.5 *μ*M MIA; blue, 3.0 *μ*M MIA. (c) Representative flow cytometry scatter plots of double-positive staining for active caspase-1 and propidium iodide (PI). (d) Double-positive rate for active caspase-1 and PI after 12 h, assessed by flow cytometry. (e) Extracellular ATP concentration of chondrocytes measured by the luciferase reaction. (f) Representative western blot, (g) relative fold-change of intensity, and (h) mRNA expression measured by qRT-PCR for P2X7, MMP13, collagen II, and IL-1*β* level normalized to the control. Data are presented as means ± SD of at least three independent experiments. ^∗^*p* < 0.05, ^∗∗^*p* < 0.01.

The results of the CCK-8 assay revealed that treatment with MIA decreased cell viability in a dose- and time-dependent manner (Figure 1(a)). Chondrocyte viability was significantly lower after treatment with 1.5 *μ*M MIA for 12 h than that in the control. Cell viability after 12 h was 81.70% in the 0.75 *μ*M MIA group, 71.24% in the 1.5 *μ*M MIA group, and 62.07% in the 3 *μ*M MIA group, compared with the control. Treatment with MIA for 24 h caused too much chondrocyte damage, while 12 h MIA treatment sufficiently induced significant differences. Therefore, MIA (0, 0.75, 1.5, and 3 *μ*M) treatment for 12 h was used in subsequent experiments.

MIA also increased LDH release in a dose-dependent manner, with a significant difference observed from the control when the chondrocytes were treated with 1.5 *μ*M MIA, suggesting inflammation (Figure 1(b)). Furthermore, the double-positive staining rate of activated caspase-1 and PI increased significantly, by 12.63% and 17.00%, in the 1.5 *μ*M and 3 *μ*M MIA groups, respectively (Figures 1(c) and 1(d)), indicating that pyroptosis was the cause of inflammation.

Elevated ATP levels activate P2X7R, triggering cell inflammation [19]. In this study, extracellular ATP concentration was positively correlated with MIA concentration (Figure 1(e)). When treated with 1.5 *μ*M MIA, the ATP content in the cell culture supernatant was significantly higher than that in the control group, suggesting appropriate conditions to activate P2X7. Western blotting and qRT-PCR analysis revealed that 1.5 *μ*M MIA significantly increased MMP13, P2X7, and IL-1*β* expression, but significantly decreased collagen II expression, in a dose-dependent manner (Figures 1(f) and 1(h)). Therefore, 1.5 *μ*M MIA treatment for 12 h was used for subsequent experiments. Quantified protein expression data are shown in Figure 1(g). Collectively, these results indicated that MIA activated P2X7 and triggered a pyroptotic inflammatory response.

### 3.2. Gain and Loss of P2X7 Function Affects Extracellular Matrix Degradation and IL-1*β* Release

**Figure 2:**
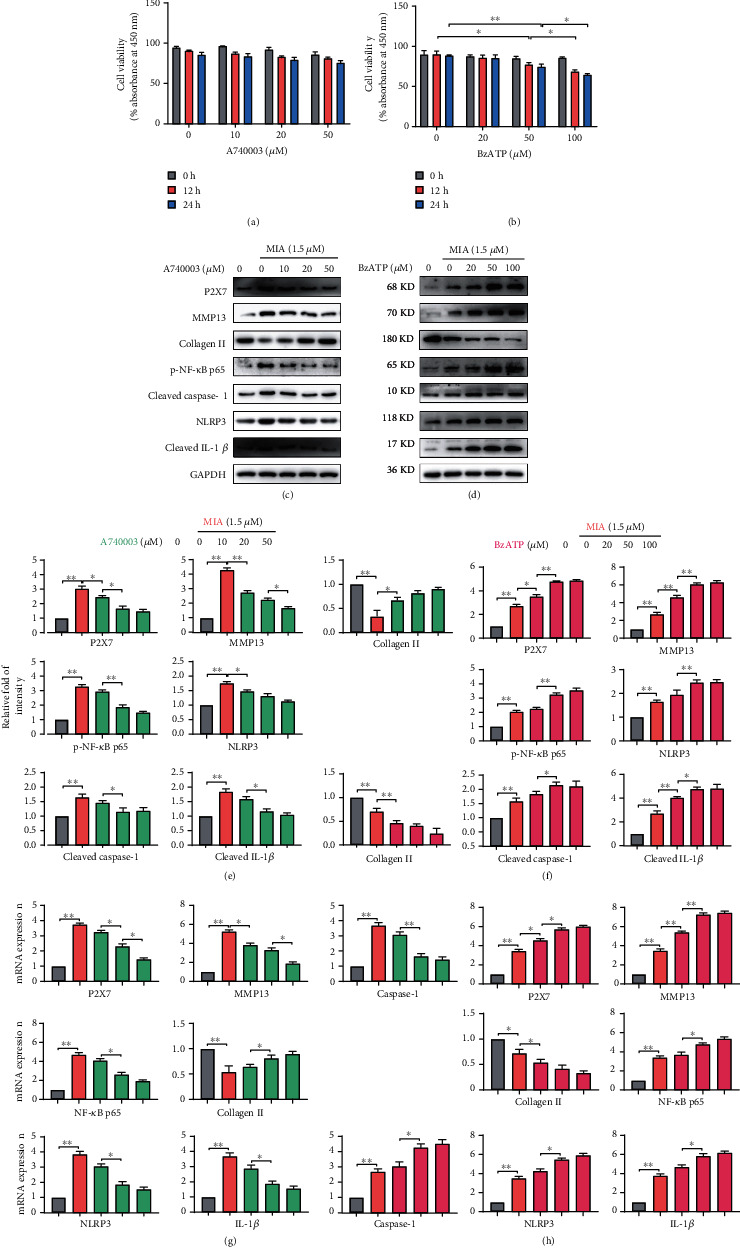
A740003 and BzATP regulate P2X7 function. Cell viability was measured for chondrocytes treated with (a) A740003 (10, 20, and 50 *μ*M) or (b) BzATP (10, 50, and 100 *μ*M) for 12 or 24 h. (c, d) Representative western blots, (e, f) relative fold-change of intensity, and (g, h) mRNA expression measured by qRT-PCR for P2X7, MMP13, caspase-1, collagen II, NK-*κ*B p65, and IL-1*β* level for chondrocytes treated for 12 h with A740003 and BzATP. Grey, 0 *μ*M MIA; orange, 1.5 *μ*M MIA; green, 1.5 *μ*M MIA+10/20/50 *μ*M A740003; red, 1.5 *μ*M MIA+20/50/100 *μ*M BzATP. Data are presented as means ± SD of at least three independent experiments. ^∗^*p* < 0.05, ^∗∗^*p* < 0.01.

Because MMP13 promotes extracellular matrix degradation in chondrocytes, while IL-1*β* induces an OA-like inflammatory response [20], we chose P2X7R antagonist A740003 and agonist BzATP to determine whether P2X7R activation or inhibition affected the expression of these effector molecules. According to related research [21], we employed concentration gradients of 0, 10, 20, and 50 *μ*M A740003 and 0, 20, 50, and 100 *μ*M BzATP. The CCK-8 assay revealed no difference in cell viability after A740003 treatment (Figure 2(a)). However, BzATP treatment decreased cell viability in a dose- and time-dependent manner (Figure 2(b)). Treatment with BzATP for 24 h caused excessive chondrocyte damage, and cell viability was significantly lower after 50 *μ*M BzATP treatment for 12 h than that in the control. Therefore, A740003 and BzATP treatments for 12 h were used in subsequent experiments.

Western blotting and qRT-PCR analysis revealed that BzATP treatment increased P2X7, MMP13, caspase-1, IL-1*β*, NF-*κ*B p65, and NLRP3 expression but decreased collagen II expression (Figures 2(d) and 2(h)). In contrast, A740003 inhibited P2X7, leading to the opposite expression patterns (Figures 2(c) and 2(g)). Treatment with 50 *μ*M BzATP or 20 *μ*M A740003 caused significant differences in the expression of these indicators compared with that in the control group. Therefore, we chose 12 h treatment with 20 *μ*M A740003 and 50 *μ*M BzATP for subsequent experiments. Combined with the quantified expression protein data (Figures 2(e) and 2(f)), the results suggested that A740003 and BzATP regulated P2X7, influencing its ability to promote extracellular matrix degradation and IL-1*β* release.

### 3.3. Inhibition of NF-*κ*B and NLRP3 Rescues P2X7-Mediated Extracellular Matrix Degradation and IL-1*β* Release

**Figure 3:**
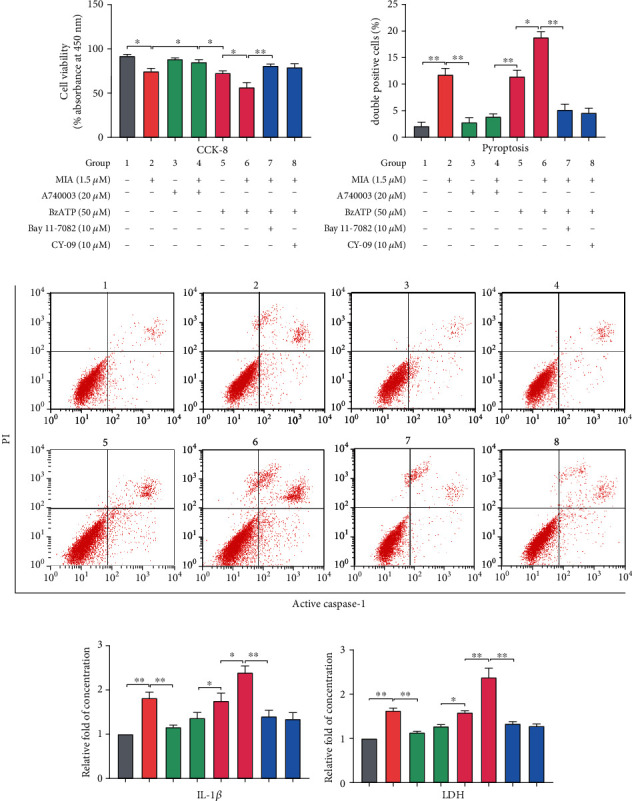
NF-*κ*B or NLRP3 inhibitors attenuate pyroptotic inflammation. (a) Cell viability of chondrocytes treated with MIA (1.5 *μ*M) with or without A740003 (20 *μ*M), BzATP (50 *μ*M), BzATP+Bay 11-7082 (10 *μ*M), or BzATP+CY-09 (10 *μ*M) for 12 h. (b) Double-positive staining rate for activated caspase-1 and propidium iodide (PI). (c) Scatter plot evaluating cell viability measured by flow cytometry. (d) ELISA analysis of extracellular inflammatory factor IL-1*β*. (e) Cell damage analyzed by LDH assay. Data are presented as means ± SD of at least three independent experiments. ^∗^*p* < 0.05, ^∗∗^*p* < 0.01.

The NF-*κ*B signaling pathway affects OA pathobiology and progression by enhancing MMP13 transcription and decreasing collagen II expression [20]. Furthermore, NF-*κ*B interacts with NLRP3 to activate caspase-1 and promote IL-1*β* release [22]. We observed increased NF-*κ*B p65 and NLRP3 expression in chondrocytes after P2X7R activation (Figures 2(f) and 2(d)). Treatment with A740003 mitigated the MIA-induced decrease in cell viability, whereas BzATP treatment further decreased cell viability. Treatment with NF-*κ*B or NLRP3 inhibitors rescued chondrocyte viability in the MIA- and BzATP-treated groups (Figure 3(a)). Flow cytometry (Figures 3(b) and 3(c)) and ELISA (Figure 3(d)) revealed that NF-*κ*B or NLRP3 inhibitors reduced the number of double-positive (activated caspase-1 and PI) cells and IL-1*β* release in the MIA and BzATP-treated groups, respectively. The results of LDH concentration measurement (Figure 3(e)) were consistent with those of the CCK-8 assay, ELISA, and flow cytometry, indicating chondrocyte damage.

**Figure 4:**
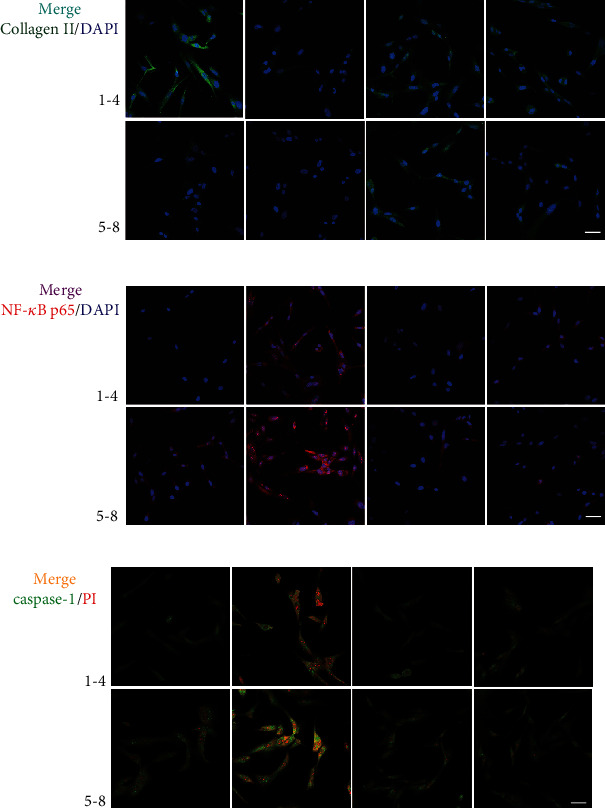
Immunofluorescence staining of (a) collagen II (green), (b) NF-*κ*B p65 (red), and (c) caspase-1/PI (green/red) immunoreactivity (scale bar: 50 *μ*m). Nuclei were stained with DAPI (blue). Groupings are the same as shown in Figure 3.

**Figure 5:**
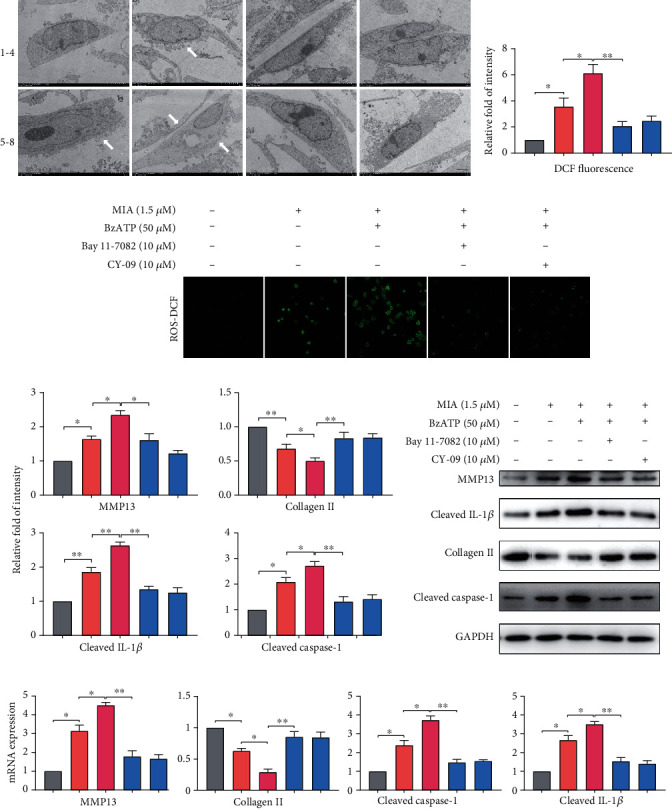
Inhibition of NF-*κ*B and NLRP3 changes cellular morphology and reduces ROS production and inflammatory factor release. (a) Representative TEM images of cellular morphology changes (white arrows) such as cell swelling, cell membrane protrusion, and cell nucleus atrophy (scale bar: 5 *μ*m). Groupings are the same as described in Figure 3. (b, c) Reactive oxygen species levels in cells analyzed by DCF fluorescence intensity. (f) qRT-PCR detection of MMP13, collagen II, P2X7, and IL-1*β* mRNA levels normalized to the control. (d, e) Representative western blots of protein expression levels. GAPDH was used as the control. Data are presented as means ± SD of at least three independent experiments. ^∗^*p* < 0.05, ^∗∗^*p* < 0.01.

Immunofluorescence analysis confirmed that MIA treatment significantly increased NF-*κ*B p65 nuclear translocation and caspase-1/PI uptake, while decreasing collagen II expression (Figures 4(a)–4(c)). However, treatment with A740003 and the NF-*κ*B and NLRP3 inhibitors significantly reversed MIA-induced damage, while BzATP treatment aggravated it. The unmerged pictures are shown in Figure S3. Treatment with MIA and BzATP increased cell welling, cell membrane protrusion, and cell nucleus atrophy (Figure 5(a)). In contrast, treatment with A740003 and the NF-*κ*B and NLRP3 inhibitors caused few morphological changes compared with that of the control. Notably, P2X7 activation triggered ROS production (Figures 5(b) and 5(c)) and expression and release of inflammatory molecules (Figures 5(e) and 5(f)) in the MIA-treated groups, regardless of BzATP treatment. Quantified protein expression data are shown in Figure 5(d). Accordingly, NF-*κ*B inhibition restored collagen II expression to control levels, as did NLRP3 inhibition. Thus, these inhibitors suppressed extracellular matrix degradation and IL-1*β* release, suggesting that P2X7 induces OA-like pyroptotic inflammation via NF-*κ*B and NLRP3 crosstalk.

### 3.4. P2X7 Increases Cartilage Loss and Bone Resorption in OA Rats

**Figure 6:**
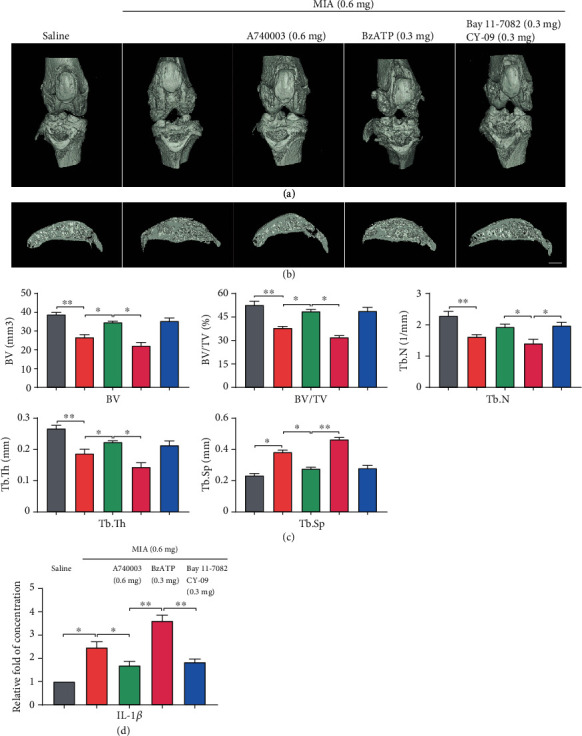
MIA-induced OA evaluated by micro-CT. (a) Frontal views of the knee joints 4 weeks after MIA injection, represented as three-dimensional micro-CT images. (b) Sagittal views of medial compartment subchondral bone. (c) Quantification of bone morphological parameters (BV, BV/TV, Tb.Sp, Tb.N, and Tb.Th). (d) ELISA analysis of IL-1*β* in joint-cavity-lavage fluid. Data are presented as means ± SD of at least three independent experiments. ^∗^*p* < 0.05, ^∗∗^*p* < 0.01. Scale = 1 mm.

Increased osteolysis in the tibial subchondral bone indicated that MIA or BzATP injection induced significant bone resorption. Nonetheless, A740003 or inhibitor (Bay 11-7082+CY-09) injections reduced bone loss, while increasing knee-joint integrity and subchondral bone mass, indicating diminished osteolysis (Figures 6(a) and 6(b)). Subchondral BV in the tibial plateau was 0.27 ± 0.03 mm^3^ in the MIA group, which was significantly lower than that in the control (0.39 ± 0.04 mm^3^). In rats treated with A740003, MIA+BzATP, or MIA+BzATP+inhibitors, BV was 0.35 ± 0.04, 0.22 ± 0.05, and 0.35 ± 0.03 mm^3^, respectively. All bone parameters (BV, BV/TV, Tb.N, Tb.Th, and Tb.Sp) demonstrated that A740003 and the inhibitors suppressed bone destruction in vivo (Figure 6(c)). ELISA (Figure 6(d)) revealed that the NF-*κ*B or NLRP3 inhibitor reduced the IL-1*β* content in the synovial fluid of the joint cavity in the MIA- and BzATP-treated groups.

### 3.5. P2X7 Exacerbates OA Progression In Vivo

**Figure 7:**
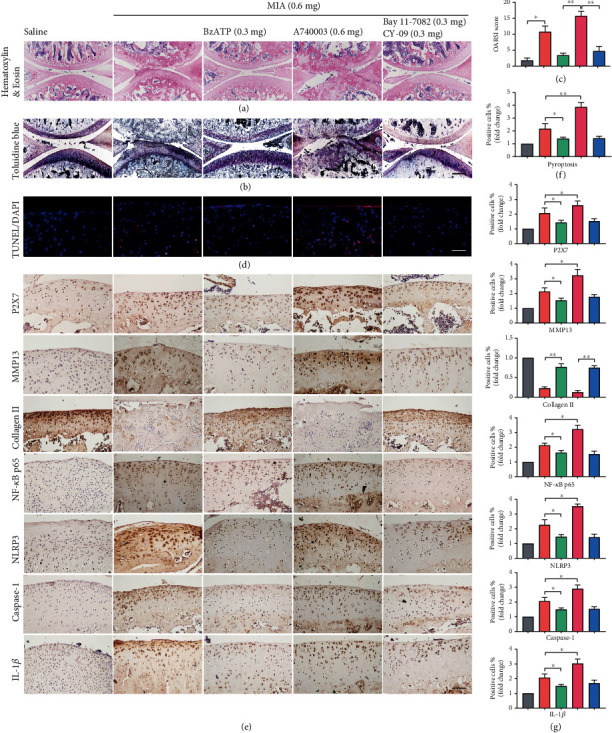
Staining evaluations of inhibitor treatments and P2X7 activation in knee-joint cartilage of MIA-induced OA rats. Cartilage and subchondral bone of knee-joints were stained using (a) H&E and (b) Toluidine Blue O (scale bar: 500 *μ*m). (c) Quantitative analysis of OARSI score from (a, b). (d) TUNEL staining of sagittal central sections of the cartilage in each group (scale bar: 50 *μ*m). (f) Quantitative data for (d). (e) IHC staining of P2X7, MMP13, collagen II, NF-*κ*B p65, NLRP3, IL-1*β*, and caspase-1 (scale bar: 50 *μ*m). (g) Quantitative analysis of immune-positive cells. Data are presented as means ± SD of at least three independent experiments. ^∗^*p* < 0.05, ^∗∗^*p* < 0.01.

Staining with H&E and Toluidine Blue O (Figures 7(a) and 7(b)) indicated that P2X7 activation induced collagen loss and decreased articular-cartilage thickness. However, A740003 and inhibitor treatment significantly inhibited cartilage degradation in OA-induced rats (Figure 7(c)). As expected, TUNEL (Figure 7(d)) and IHC staining (Figure 7(e)) revealed that BzATP treatment increased P2X7, MMP13, NF-*κ*B p65, NLRP3, IL-1*β*, and caspase-1 expression, thus, accelerating chondrocyte death (Figure 7(f)). However, treatment with inhibitors reversed the OA-like phenotype, including articular cartilage loss and collagen reduction (Figure 7(g)). The unmerged pictures are shown in Figure S4. Overall, our results demonstrated that P2X7 exacerbated OA progression in vivo and inhibitors attenuated this effect.

### 3.6. Underlying Mechanisms of P2X7 in Cartilage Degradation and Pyroptotic Inflammation

**Figure 8:**
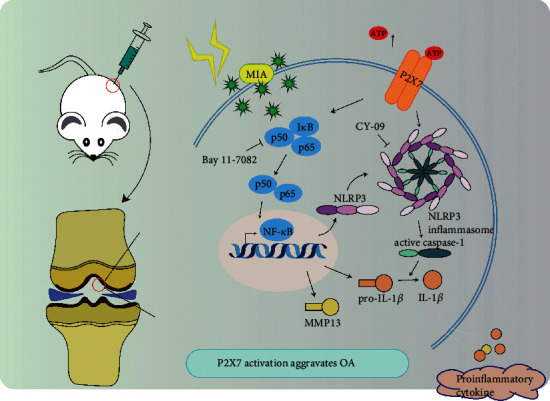
Schematic of the working hypothesis of P2X7 in OA.

Figure 8 comprehensively illustrates the experimental results from both macro- and microperspectives. The left part of the figure represents the animal experiment, in which reagents were injected into the knee-joint cavities of SD rats. The right part of the figure demonstrates the effect of the various reagents on chondrocytes and the corresponding molecular mechanism. First, MIA stimulation caused elevated extracellular ATP concentration of chondrocytes. Then, ATP activated P2X7, which, in turn, activated the NF-*κ*B and NLRP3 signaling pathways. NF-*κ*B increased MMP13 expression and collagen II degradation, while activating transcription of NLRP3 and pro-IL-1*β*. At the same time, NLRP3-dependent caspase-1 cleavage led to maturation of IL-1*β*, and bioactive IL-1*β* was released to the outside of the cell, further stimulating activation of NF-*κ*B and aggravating OA. Therefore, P2X7 activation mediated extracellular matrix degradation and pyroptotic inflammation in chondrocytes via NF-*κ*B/NLRP3 crosstalk.

## 4. Discussion

Maintaining chondrocyte balance and reducing cartilage degeneration are critical to alleviating OA development and progression. As a key inflammation switch, purinergic receptor P2X7R has recently received attention as a potential therapeutic target for OA [23]. Here, we demonstrated that P2X7 activation promoted extracellular matrix degradation and pyroptotic inflammation of chondrocytes through NF-*κ*B/NLRP3 crosstalk, thus, aggravating OA.

Extracellular matrix degradation and the release of inflammatory factors are the main factors that cause chondrocyte imbalance. The interference of anabolic and catabolic activities can cause negative cellular changes and even death [24–27]. Dying cells and damaged tissues release large amounts of ATP, which is a critical signal for P2X7 activation [28]. Extracellular ATP concentrations measured at the inflammation site are extremely high, reaching hundreds of micromolar, in contrast to the low nanomolar range found in healthy tissues. Research using rat spinal fluid showed that MIA induces increased ATP and microglial P2X7 expression, resulting in joint pain [29]. In addition, MIA induces chondrocyte changes that produce an OA-like phenotype [15]. However, prior to this study, we had little data regarding MIA-induced activation of P2X7 resulting from ATP release from chondrocytes, leading to the development of a high-ATP extracellular environment. Here, we found that as MIA concentration increased, cell activity decreased, LDH release increased, and the number of caspase-1/PI double-positive cells increased, thus, indicating negative effects on cell proliferation and elevated pyroptosis. We also observed increased ATP concentration and expression of P2X7 and inflammation-related indicators. These findings are consistent with those of previous studies suggesting that P2X7 may be involved in ATP release from chondrocytes [30]. Thus, taken together, the data confirmed that MIA induces chondrocytes to release ATP, activate P2X7, and produce an OA-like pyroptosis phenotype.

Further research is necessary to definitively conclude that P2X7 activation is responsible for extracellular matrix degradation and pyroptotic inflammation. Previous studies determined that P2X7R antagonists and agonists influence P2X7 expression and activation. For example, AZD9056 inhibited P2X7 to relieve MIA-induced joint swelling and inflammatory-factor release in Wistar rats [31]. Continuous A740003 blockade of P2X7 suppressed MIA-induced mechanical allodynia [29], while BzATP was shown to be a potent and selective P2X7 activator [28]. Interestingly, we found that A740003 alone did not affect chondrocyte activity but that BzATP and cell activity were inversely correlated. When cells were treated with MIA and A740003, P2X7, MMP13, and IL-1*β* expression decreased, while collagen II expression increased. The opposite effect occurred with BzATP. These results confirmed that P2X7 activation promotes extracellular matrix degradation and inflammatory-factor release, while inhibiting P2X7 attenuates these effects. A combination of metabolic and mechanical factors is thought to influence cartilage degradation [32, 33]. These factors activate the NLRP3 inflammasome and then NF-*κ*B, followed by upregulation of IL-1*β* and TNF-*α*, which are the main cartilage-degrading cytokines in OA [34]. NF-*κ*B and NLRP3 are interconnected inflammatory pathways that cross talk to promote the release of inflammatory factors, leading to pyroptosis [22]. Here, we showed for the first time that the NF-*κ*B and NLRP3 signaling pathways are involved in P2X7-mediated extracellular matrix degradation and pyroptotic inflammation. When cells were treated with MIA and A740003, NF-*κ*B p65, NLRP3, and caspase-1 expression decreased, while BzATP caused the opposite effect. Thus, P2X7 affects downstream NF-*κ*B and NLRP3 activity.

P2X7 is known to aggravate OA in the mouse knee joint through activation of NF-*κ*B and upregulate the expression of inflammatory mediators, such as MMP13 [31]. Our previous research found that moderate mechanical stimulation could inhibit NF-*κ*B and cell pyroptosis to alleviate OA [35], and treatment with loganin and ursolic acid had similar effects [36, 37]. To further confirm that P2X7 activation aggravated OA symptoms through interaction with the NF-*κ*B and NLRP3 pathways, we used NF-*κ*B and NLRP3 inhibitors in several experiments. The selective NF-*κ*B inhibitor Bay 11-7082 inhibits I*κ*B*α* degradation, thus, preventing nuclear translocation and transcriptional activity of p65-containing complexes [38]. Additionally, CY-09 directly inhibits NLRP3 ATPase activity via binding to the NACHT domain, thus, resulting in the termination of NLRP3 inflammasome assembly [39]. Treating OA cells with Bay 11-7082 or CY-09 increased cell activity, decreased LDH release, decreased ROS production, decreased the number of caspase-1/PI double-positive cells, and improved cell morphology, indicating the restoration of cell proliferation and amelioration of pyroptosis. Treatment with these inhibitors also reduced the inflammatory response, as demonstrated by the decreased IL-1*β* content in the cell supernatant and IALF. Treating chondrocytes with MIA and A740003 had a similar effect, which was confirmed in vivo. Our result suggests that inhibiting the NF-*κ*B pathway could inhibit the NLRP3 pathway and vice versa. When NF-*κ*B and NLRP3 inhibitors were used in combination, micro-CT and IHC staining demonstrated a significant reduction of cartilage degradation and bone destruction. Therefore, the NF-*κ*B and NLRP3 signaling pathways form a crosstalk connection to promote P2X7-induced chondrocyte extracellular matrix degradation and pyroptotic inflammation.

Our study opens the door to many new avenues of research. Although bioinformatics analyses have shown that P2X7 is highly expressed in the knee-joint tissues of patients with OA, additional clinical data are necessary to support the conclusions reached in this study. Furthermore, our current experimental model can be improved for greater applicability to the human body. Finally, in-depth experiments are warranted to elucidate the precise mechanisms of P2X7 action.

## 5. Conclusions

We clarified the direct effect of P2X7 on OA progression. To our knowledge, this is the first study to demonstrate that P2X7 activation promotes extracellular matrix degradation and pyroptotic inflammation caused by MIA-induced elevation of extracellular ATP. This is also the first study to establish that NF-*κ*B/NLRP3 crosstalk mediates P2X7 activation in OA. The use of a P2X7 inhibitor and related pathway inhibitors effectively alleviated the inflammation caused by P2X7 activation, suggesting a possible treatment path for OA, and indicated the potential of P2X7 as a drug target in the treatment of inflammation. The emerging role of P2X7 in chondrocyte inflammation may help develop effective and targeted drugs and inhibitors, thus, providing a new therapeutic option to alleviate OA symptoms.

## Data Availability

All original data are available from the corresponding author upon request. Source data for Figures 1, 2, and 5 are provided with the paper.

## Supplementary Materials

Supplementary Materials Figure S1: differentially expressed genes and P2X7 expression. (a) Volcano plots were constructed using fold change and *p* values, demonstrating varied mRNA expression between healthy and OA cartilage samples. (b) Expression of P2X7 in healthy and OA human knee cartilage tissues. Figure S2: rat primer sequences. Figure S3: unmerged pictures from Figure 4. Immunofluorescence analyses confirmed that MIA treatment significantly increased NF-*κ*B p65 nuclear translocation and caspase-1/PI uptake, while decreasing collagen II expression. However, treatment with A740003, Bay 11-7082 (NF-*κ*B inhibitor), and CY-09 (NLRP3 inhibitor) significantly reversed MIA-induced damage, while BzATP treatment aggravated it (scale bar: 50 *μ*m). Figure S4: unmerged pictures from Figure 7(d). As expected, TUNEL staining revealed that BzATP treatment accelerated chondrocyte death. However, treatment with inhibitors reversed the OA-like phenotype (scale bar: 50 *μ*m).
