# Supplementary material for: P2X7 Receptor Induces Pyroptotic Inflammation and Cartilage Degradation in Osteoarthritis via NF-κB/NLRP3 Crosstalk
Source: Oxid Med Cell Longev. 2021 Jan 16;2021:8868361. doi: 10.1155/2021/8868361 (PMC7834826; doi:10.1155/2021/8868361)
Supplement: Supplementary Materials — Figure S1: differentially expressed genes and P2X7 expression. (a) Volcano plots were constructed using fold change and p values, demonstrating varied mRNA expression between healthy and OA cartilage samples. (b) Expression of P2X7 in healthy and OA human knee cartilage tissues. Figure S2: rat primer sequences. Figure S3: unmerged pictures from Figure 4. Immunofluorescence analyses confirmed that MIA treatment significantly increased NF-κB p65 nuclear translocation and caspase-1/PI uptake, while decreasing collagen II expression. However, treatment with A740003, Bay 11-7082 (NF-κB inhibitor), and CY-09 (NLRP3 inhibitor) significantly reversed MIA-induced damage, while BzATP treatment aggravated it (scale bar: 50 μm). Figure S4: unmerged pictures from Figure 7(d). As expected, TUNEL staining revealed that BzATP treatment accelerated chondrocyte death. However, treatment with inhibitors reversed the OA-like phenotype (scale bar: 50 μm). [file 8868361.f1.docx]

**Supplementary Materials**

**P2X7 receptor induces pyroptotic inflammation and cartilage degradation in osteoarthritis via NF-κB/NLRP3 crosstalk**

Zihao Li^1^, Ziyu Huang^2^, He Zhang^1^, Jinghan Lu^1^, Yicheng Tian^1^, Yingliang Wei^1^, Yue Yang^1^, and Lunhao Bai^1^

^1^ Department of Orthopedic Surgery, Shengjing Hospital of China Medical University, Shenyang 110000, China

^2^ Foreign Languages College, Shanghai Normal University, Shanghai 200234, China

Correspondence: Lunhao Bai; lunhaobai_ace@163.com

**Figure S1: Volcano plots of the differentially expressed genes and scatter plot of P2X7**


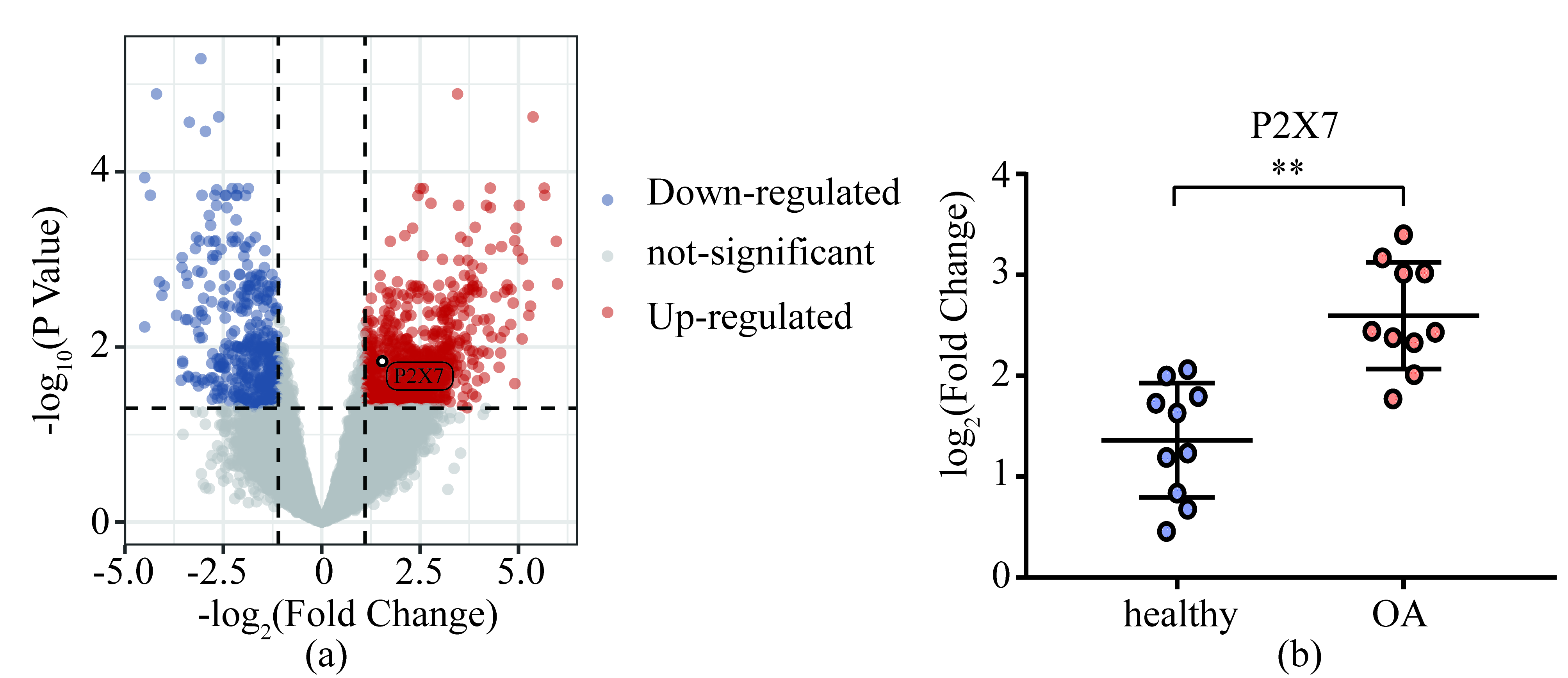


(a) Volcano plots were constructed using fold change and *p* values and showed varied mRNA expression between the healthy and OA cartilage samples. (b) The expression of P2X7 in healthy and OA human knee cartilage tissues. ⁎*p* < 0.05, ⁎⁎*p* < 0.01.

**Figure S2: Rat primer sequences**

| Gene Name | Species |  | Sequence |
| --- | --- | --- | --- |
| P2X7 | rat | forward | 5´- TTAGTACACGGCATCTTCGACACG - 3´ |
|  |  | reverse | 5´- AGCTTCTGTTCTTGGCCTTCTGAC - 3´ |
| MMP13 | rat | forward | 5´- TGGTCCAGGAGATGAAGACC - 3´ |
|  |  | reverse | 5´- GTGCAGACGCCAGAAGAATC - 3´ |
| collagen II | rat | forward | 5´- ACGCTCAAGTCGCTGAACAACC -3’ |
|  |  | reverse | 5´- ATCCAGTAGTCTCCGCTCTTCCAC -3’ |
| NF-κB p65 | rat | forward | 5´- GGCTTCTATGAGGCTGAACTCTGC - 3´ |
|  |  | reverse | 5´- CTTGCTCCAGGTCTCGCTTCTTC - 3´ |
| IL-1β | rat | forward | 5´- ACAGCAGCATCTCGACAAGAGC - 3´ |
|  |  | reverse | 5´- CCACGGGCAAGACATAGGTAGC - 3´ |
| caspase-1 | rat | forward | 5’- TTTCCGCAAGGTTCGATTTTCA - 3’ |
|  |  | reverse | 5’- GGCATCTGCGCTCTACCATC - 3’ |
| NLRP3 | rat | forward | 5’- CCTGGGGGACTTTGGAATCAG - 3’ |
|  |  | reverse | 5’- GATCCTG ACAACACGCGGA - 3’ |
| GAPDH | rat | forward | 5´- GGCACAGTCAAGGCTGAGAATG - 3´ |
|  |  | reverse | 5´- ATGGTGGTGAAGACGCCAGTA - 3´ |

**Figure S3: Unmerged pictures in Figure 4**


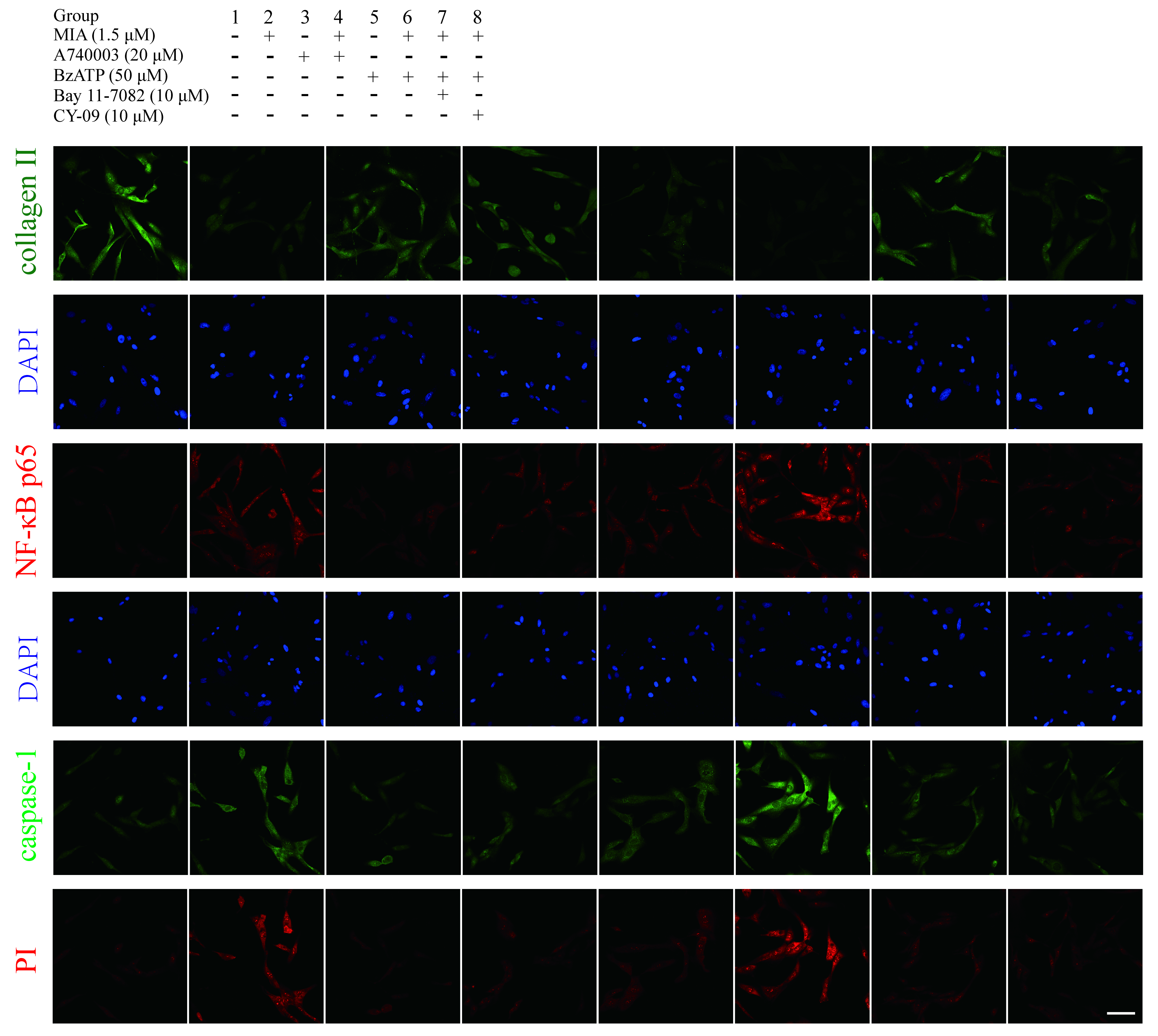


Immunofluorescence analyses confirmed that MIA significantly increased NF-κB p65 nuclear translocation and caspase-1/PI uptake, while decreasing collagen II expression. However, A740003, BAY 11-7082 (NF-κB inhibitor), and CY-09 (NLRP3 inhibitor) significantly reversed MIA-induced damage, while BzATP aggravated it (scale bar: 50 µm).

**Figure S4: Unmerged pictures in Figure 7(d)**


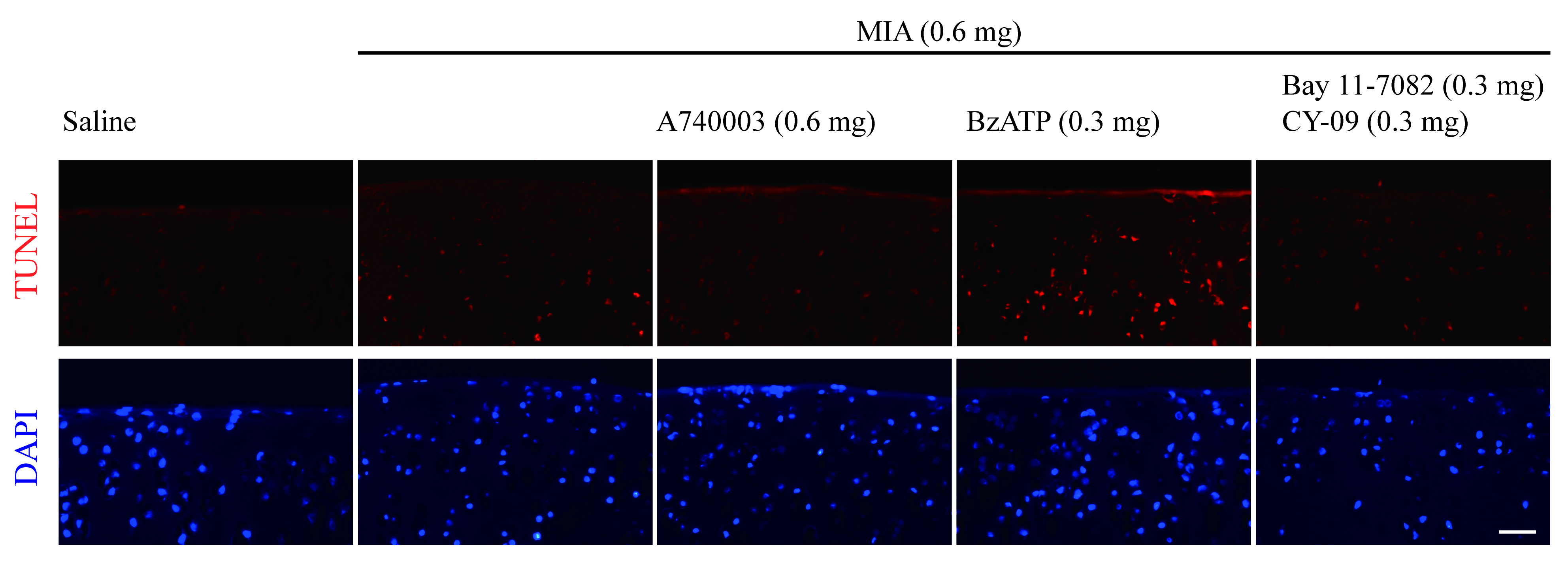


As expected, TUNEL staining revealed that MIA and BzATP accelerated chondrocyte death. However, inhibitors reversed OA-like phenotypes (scale bar: 50 µm).
